# Natural Killer Cell Response to Chemotherapy-Stressed Cancer Cells: Role in Tumor Immunosurveillance

**DOI:** 10.3389/fimmu.2017.01194

**Published:** 2017-09-25

**Authors:** Alessandra Zingoni, Cinzia Fionda, Cristiana Borrelli, Marco Cippitelli, Angela Santoni, Alessandra Soriani

**Affiliations:** ^1^Department of Molecular Medicine, Sapienza University of Rome, Laboratory Affiliated to Istituto Pasteur Italia – Fondazione Cenci Bolognetti, Rome, Italy; ^2^Center for Life Nano Science@Sapienza, Istituto Italiano di Tecnologia, Rome, Italy; ^3^Neuromed I.R.C.C.S. – Istituto Neurologico Mediterraneo, Pozzilli, Italy

**Keywords:** natural killer cells, immunochemotherapy, cancer, stress, natural killer cell activating ligands, damage-associated molecular patterns, death receptors, PDL-1

## Abstract

Natural killer (NK) cells are innate cytotoxic lymphoid cells that actively prevent neoplastic development, growth, and metastatic dissemination in a process called cancer immunosurveillance. An equilibrium between immune control and tumor growth is maintained as long as cancer cells evade immunosurveillance. Therapies designed to kill cancer cells and to simultaneously sustain host antitumor immunity are an appealing strategy to control tumor growth. Several chemotherapeutic agents, depending on which drugs and doses are used, give rise to DNA damage and cancer cell death by means of apoptosis, immunogenic cell death, or other forms of non-apoptotic death (i.e., mitotic catastrophe, senescence, and autophagy). However, it is becoming increasingly clear that they can trigger additional stress responses. Indeed, relevant immunostimulating effects of different therapeutic programs include also the activation of pathways able to promote their recognition by immune effector cells. Among stress-inducible immunostimulating proteins, changes in the expression levels of NK cell-activating and inhibitory ligands, as well as of death receptors on tumor cells, play a critical role in their detection and elimination by innate immune effectors, including NK cells. Here, we will review recent advances in chemotherapy-mediated cellular stress pathways able to stimulate NK cell effector functions. In particular, we will address how these cytotoxic lymphocytes sense and respond to different types of drug-induced stresses contributing to anticancer activity.

## Introduction

Natural killer (NK) cells represent a crucial component of antitumor innate immune response displaying cytotoxic functions and secreting several cytokines/chemokines (1, 2).

Natural killer cell cytotoxic activity regulation depends on an integrated interplay between inhibitory receptors and numerous activating receptors acting in concert to efficiently eliminate tumor cells.

Relevant activating receptors for tumor cell recognition are NKG2D that recognizes MICA/B and ULBPs proteins, orthologs of the mouse RAE1 molecules, DNAM-1 that binds two ligands named poliovirus receptor (PVR/CD155) and Nectin-2 (CD112), and the receptors NKp30, NKp44, and NKp46 belonging to the natural cytotoxicity receptors and shown to interact with a broad spectrum of ligands (3).

Natural killer cells also express inhibitory receptors for molecules of the major histocompatibility complex (MHC) class I, which are Ly49 receptors in mice, *k*iller cell *i*mmunoglobulin-like *r*eceptors (KIRs) that bind to HLA-A, -B, and -C molecules in humans, and the CD94-NKG2A heterodimer in both species (4). In addition, NK cells express two inhibitory receptors for PVR, called TACTILE (CD96) and TIGIT, that counterbalance the DNAM-1-mediated activation of NK cells (5).

The activation of NK cells leads to the release of cytotoxic granules containing perforin and various granzymes and to cytokine production, most prominently interferon-γ (IFN-γ) (6–8). In addition, the expression at the cell surface of death-inducing ligands belonging to the tumor necrosis factor (TNF) family, such as Fas ligand (FasL) and TNF-related apoptosis-inducing ligand (TRAIL), also drives the activation of the caspase enzymatic cascade through the binding to the death receptors (DRs), namely, Fas, DR4 (TRAIL-RI), and DR5 (TRAIL-RII), on target cells (9, 10).

More recently, immunological checkpoint molecules commonly associated with T cells, such as CTLA-4 and PD-1, have been described on NK cells as negative regulators of their immune function (11–13).

Conventional chemotherapies were initially designed to produce antiproliferative or cytotoxic effects on dividing tumor cells. However, as result of numerous demonstrations indicating that an endogenous antitumor immunity is essential for complete remission during tumor therapy (14–16) several antineoplastic drugs, even at low doses, have been reconsidered also as potential immunomodulatory agents (17).

In this context, it has becoming always more evident that dying or stressed cells release or expose stress molecules, called damage-associated molecular patterns (DAMPs) that can alert the immune system (18). Moreover, many chemotherapy-mediated stress pathways modulate the expression of NK cell activating and inhibitory ligands, rendering tumor cells more immunogenic.

In this review, we will summarize the effects of different chemotherapeutic agents on the activity of NK cells, emphasizing the immunomodulatory effects of both conventional and low concentrations of drugs at the interface between stressed or dying cancer cells and the immune system, in the attempt of exploiting them for therapeutic purposes.

## Regulation of NK Cell-Activating and -Inhibitory Ligand Expression by Chemotherapeutic Drugs

A number of evidence indicate that chemotherapy-induced sensitization of tumor cells to immune effectors plays an important role in anticancer therapy. Indeed, different types of drug-induced stresses can modulate the expression of NK cell-activating/or -inhibitory ligands on cancer cells thus affecting their recognition and elimination by NK cells (Table 1). Besides genotoxic drugs or radiotherapy, many other pharmacological compounds already approved for the treatment of different malignancies or entered in clinical trials have been described to increase NK cell-activating ligand expression (19–27). Moreover, most of these drugs are also able to downregulate NK cell-inhibitory ligand expression, so that different and multiple mechanisms concur to make tumor cells more susceptible to NK cell-mediated lysis (28–32).

**Table 1:** Chemotherapy-induced pathways and molecular targets able to modulate natural killer (NK) cell activating ligands and PDL-1 on cancer cells.

Class of chemotherapeutic agent	Pathway/molecular target	Ligand	NK cell cytotoxicity	Cancer cell type	Reference
**Proteasome inhibitor**					
Bortezomib	DNA damage response (DDR)	MICA	nd	Multiple myeloma (MM)	(24)
*Low doses*: 0.75–10 nM	nd	MICA/B, PVR, Nec-2	+	MM	(52)
	nd	MICA/B ULBP1–3, PVR, Nec-2	nd	MM	(22)
	nd	MICA/B	+	Hepatocellular carcinoma	(114)
**Histone deacetylase inhibitors**					
*Low dose*: valproic acid (1 mM)	nd	MICA/B	+	Hepatocellular carcinoma	(41)
	ERK	MICA/B, ULBP2	+	MM	(45)
	PI3K/Akt	MICA/B	+	Pancreatic cancer	(40)
Trichostatin A	HDAC1/MICA promoter	MICA/B	+	Leukemia	(42)
Suberoylanilide-hydroxamic acid	miR-17-92	MICA	+	Hepatocellular carcinoma	(46)
**Genotoxic agents**					
*Low doses*: doxorubicin (0.05–3.5 μM); melphalan (1.5–22 µM)	Reactive oxygen species-dependent DDR	MICA/B, ULBP1–3, PVR, Nec-2	+	MM	(22, 34)
Cisplatin	nd	B7-H6	+	Tumor cell lines	(23)
Ara-C, aphidicolin	STING/TBK/IRF3	RAE1	nd	B cell lymphoma	(35)
**GSK inhibitors**					
*Low doses*: LiCl (10 mM), BIO (1.5 µM), SB21 (5 µM)	STAT3 inhibition	MICA	+	MM	(20)
**BET inhibitors**					
*Low dose*: JQ1 (0.5 µM)	IRF4	MICA	+	MM	(19)
	BRD4	PDL-1	nd	Lymphoma	(28)
**HSP90 inhibitors**					
*Low doses*: radicicol (2 µM), 17-AAG (1 µM)	HSR	MICA/B	+	MM	(21)
**Microtubule assembly inhibitors**					
*Low dose*: vincristine (0.05 µg/ml)	p38 MAPK	PVR, MICA, ULBP1	+	MM	(50)
Cytochalasin D Nocodazole Docetaxel	DDR Endoplasmic reticulum stress response	MICA, ULBP1–3, PVR, Nec-2, B7-H6	+	Tumor cell lines	(51)
**Immunomodulatory drugs**					
*Low dose*: lenalidomide (10 µM)	IKZF1/3, IRF4	MICA, PVR	+	MM	(47)

*Effects on an increased NK cell recognition and killing of drug-treated tumor cells are also reported (+). *Low doses* of drugs that do not affect cell vitality are indicated*.

*nd, not done*.

In the case of genotoxic drugs or DNA replication inhibitors, the mechanisms regulating the NKp30 ligand B7-H6 expression on human cancer cells remain largely unknown (23), while much evidence indicate a major role for the DNA damage response (DDR) pathway in the upregulation of the stimulatory ligands for the NKG2D and DNAM-1 immunoreceptors. In addition, ionizing radiations represent classical stimuli to induce NKG2D ligand upregulation, through the induction of the DDR (33). The activation of the kinases ATM/ATR and the production of reactive oxygen species converge on the E2F1 factor able to activate MICA, MICB, and PVR transcription on multiple myeloma (MM) cells by doxorubicin and melphalan (34). On the other hand, a different pathway governing NKG2DLs expression by chemicals known to induce genotoxic stress has been characterized in murine lymphoma cells: DDR drives to the presence of cytosolic DNA and to STING/TBK1-dependent activation of the transcription factor IRF3, responsible for the upregulation of RAE1 expression (35). Interestingly, in murine leukemia cells, concomitantly to NKG2D ligand upregulation, DDR-activating therapeutic agents cause a loss of the inhibitory NK cell ligand Clr-b, thus enhancing the cytotoxicity mediated by NKRP1B^+^ NK cells (36).

Non-lethal heat shock mimicking hyperthermia therapy can promote NKG2DL expression both in human and murine cancer cells but with different mechanisms. MICA and MICB upregulation occurs at the transcriptional level *via* HSF1 activation (37) and, with a similar mechanism, MICA and MICB expression on MM cells is enhanced by HSP90 chaperone inhibitors that activate this transcription factor (21). In a different way, increased surface expression of the mouse NKG2D ligand Mult1 depends on the inhibition of protein ubiquitination and lysosomal degradation (38).

Treatment of different tumor cell types with epigenetic drugs, like histone deacetylase inhibitors (HDACi) and DNA-methyltransferase inhibitors (DNMTi) (25–27, 39–43), leads to the upregulation of NKG2DLs and PVR surface levels, although it downregulates B7-H6 expression (44). For DNMTi the molecular mechanisms underlying NKG2DLs upregulation are still unclear, while different pathways cooperate in the regulation of these molecules in response to HDACi, and this might depend on the type of tumor and the dose of the drug used. In particular, valproic acid (VPA) has been reported to upregulate MICA/B with a mechanism dependent on PI3K/Akt pathway in pancreatic cancer cells (40), while the involvement of ERK in MICA/B and ULBP2 upregulation in response to VPA has been shown in MM cells (45). Moreover, Yang and colleagues proposed that the capability of the HDACi suberoylanilide-hydroxamic acid (SAHA) to increase MICA expression in hepatoma cancer cells is dependent on miR-17-92 cluster (46).

In MM cells, the bromodomain and extra terminal domain inhibitors (BETi) and immunomodulatory drugs (IMiDs) can block the repressive activity of the transcription factors IRF4 and IKZF1/3 on MICA and PVR promoters (19, 47). In addition, both these therapeutic agents can downregulate the expression of PD-L1 on cancer cells (28, 29, 31, 32). Indeed, BETi interrupt the activity of the epigenetic reader protein BRD4 on PD-L1 promoter region, by significantly reducing both the constitutive and IFN-γ inducible expression of this ligand. In this regard, the downstream mediators of IFN-γ signaling, JAK kinases, can be pharmacologically blocked to negatively regulate PD-L1 expression in cancer cells (48). Furthermore, drugs disrupting RAF/MEK/ERK signaling pathway, such as Sorafenib and the TLR3 agonists poly-IC, can synergistically reduce the percentage of tumor cells expressing PD-L1 and enhance NK and T cell activation in a mouse model of hepatocarcinoma (49).

Regarding drugs that disrupt the microtubule assembly, sub-lethal doses of Vincristine can activate p38 MAPK and regulate NKG2DL expression both at transcriptional and posttranscriptional level in MM cells (50). Moreover, Cytochalasin D, nocodazole, and docetaxel can enhance NKG2D, DNAM-1, and NKp30 ligands on tumor cell surface, with MICA upregulation being dependent on both DNA damage and endoplasmic reticulum (ER) stress response (51).

Different studies have been done by using proteasome inhibitors in MM cells. In this regard, low doses of bortezomib can induce the upregulation of both NKG2D and DNAM-1 ligands (22, 52, 53), and in accordance with these data, Jinushi and colleagues reported a DDR-ATM-dependent upregulation of MICA surface levels (24). On the other hand, no significant change in NKG2DL expression was observed upon bortezomib treatment by Shi and colleagues (30). Interestingly, the latter study described the capability of bortezomib to downregulate HLA class I surface expression by sensitizing MM cells to NK cell–mediated lysis (30).

Chemotherapeutic agents can also contribute to the posttranslational regulation of NK activating ligand expression by promoting the release of soluble NKG2DLs through the modulation of the expression and activity of metalloproteinases (MMP) and ADAM enzymes on cancer cells (54). Although an increased stimulation of the shedding process in response to genotoxic agents has been reported (55), some studies using different drugs describe an inhibitory effect. Indeed, gemcitabine treatment impaired ULBP2 shedding through downregulation of ADAM10 in pancreatic cancer (56). Likewise, the hypomethylating agents, azacitidine and decitabine, reduced MICA, MICB, and ULBP2 release in AML by increasing TIMP3 expression, a potent inhibitor of MMP family (57).

Thus, antitumor therapeutics can work also as activators of different “stress pathways” that enhance tumor sensitivity to NK cell cytolysis by modulating the expression of the activating and inhibitory ligands on tumor cells.

## Modulation of DRs by Cancer Therapeutic Agents

Many cancer therapeutic drugs can induce DR expression and redistribution (58) (Table 2). Several studies described a role for different types of HDACi in the upregulation of TRAIL receptors on various malignant tumor cells (59–63). In this context, SAHA and trichostatin A (TSA) were shown to increase cell-surface expression of DR4 and DR5 in human MM cell lines (64). A study from Insinga et al. showed that different DR and their ligands (i.e., TRAIL, DR5, FasL, and Fas) are upregulated by HDACi on leukemic cells, but not in the normal counterpart of hematopoietic progenitors, promoting tumor apoptosis through the activation of the DR pathway (65).

**Table 2:** Chemotherapy-induced pathways and molecular targets able to modulate death receptors (DRs) on cancer cells.

Class of chemotherapeutic agent	Pathway/molecular target	DR	Cancer cell type	Reference
**Proteasome inhibitors**				
*Low doses*: bortezomib (5–20 nM)	DNA damage response	DR5	Tumor cell lines, renal carcinoma	(66, 67)
MG132	CHOP	DR5	Prostate cancer	(71)
**Histone deacetylase inhibitors**				
Sodium butyrate	Sp1	DR5 (caspase-3 activation)	Colorectal carcinoma	(59)
Trichostatin A (TSA), suberoylanilide-hydroxamic acid (SAHA) Sodium butyrate	p53-independent mechanism	DR5 (caspase member activation)	Tumor cell lines	(60)
*Low doses*: SAHA (500 nM), TSA (50 nM)	p21, p27, E2F	DR4, DR5 (increase of proapototic Bcl-2 family members)	Multiple myeloma	(64)
VPA	nd	DR5, FAS	Leukemia	(65)
**Genotoxic agents**				
Cisplatin, mitomycin, doxorubicin, methotrexate, etoposide	p53-dependent mechanism	FAS, DR5, DR4	Tumor cell lines	(72–74, 77)
Etoposide	NF-κB	DR5	Tumor cell lines	(76)
Doxorubicin, Ara-C, etoposide	p53-independent mechanism	DR5	Leukemia cell lines	(81)

*Low doses of drugs that do not affect cell vitality are indicated*.

*nd, not done*.

A number of studies showed that bortezomib upregulated surface expression of TRAIL receptors on a variety of human tumor cell lines, enhancing their susceptibility to NK cell lysis with a mechanism mainly dependent on TRAIL (66). In another model, a bortezomib-treated murine renal carcinoma cell line is more susceptible to both NK-cell perforin/granzyme and recombinant TRAIL-mediated apoptosis, resulting in enhanced caspase-8 activity (67). Indeed, in human non-small cell lung cancer cells this drug has been shown to trigger TRAIL-induced apoptosis *via* DR5 upregulation (68). Several pieces of evidence reported that another proteasome inhibitor, namely, MG132, increases DR5 expression cooperating in establishing apoptosis in several cancer cells (69–71).

DR4 and DR5 were demonstrated to be DNA damaging-inducible and p53-regulated genes (72–76). Accordingly, many DNA damaging chemotherapeutic agents can regulate DR expression, rendering cancer cells more sensitive to DR-elicited apoptosis (74, 75, 77–81).

Altogether, these results suggest that the extrinsic apoptotic pathway has an important role in chemotherapy-induced apoptosis through the promotion of DRs-mediated recognition by cytotoxic lymphocytes. In addition, chemotherapies can promote the cell death by regulating the balance between pro- and antiapoptotic proteins toward apoptosis. Many evidence show that drugs may control the cell intrinsic apoptosis by altering Bax and Bcl-2 expression in different tumor cells (82–86).

## Chemotherapy-Induced DAMPs Alerting NK Cells

Many anticancer chemotherapies increase the immunogenic potential of cancer cells mainly through the establishment of immunogenic cell death, or other forms of non-apoptotic death, including autophagy, and the release of the so-called DAMPs, such as high-mobility group box 1 proteins (HMGB1), ATP, heat shock proteins (HSPs), and the ER chaperone calreticulin (87).

Damage-associated molecular patterns are intracellularly sequestered in normal physiological conditions, but they can be actively secreted or aberrantly exposed on the cell surface under conditions of cellular stress.

Engagement of various target receptors present on immune cells by DAMPs leads to the elicitation of a potent antitumor immunity. Mostly, DAMPs have been proposed to activate local APCs, thus promoting the adaptive immune system. For example, both HSP70 and HMGB1 boost dendritic cell (DC) maturation through toll-like receptor 4, favoring the induction of antigen-specific T cell-mediated antitumor immune responses (88, 89). Less is known about DAMP contribution to NK cell stimulation; thus, we will focus the attention on HMGB1 and HSPs, due to their ability to exert different effects on NK cell-mediated functions.

High-mobility group box 1 protein is an endogenous nuclear factor released both by activated immune cells or injured non-immune cells, and in the extracellular milieu acts as a DAMP alerting the immune system to danger and triggering immune response activation through the interaction either with multiple TLRs and the receptor for advanced glycation end products (RAGE), expressed on a variety of cells (90). In this regard, the chemotherapeutic agent cyclophosphamide has been recently shown to facilitate NK cell activation through a process involving HMGB1 release in a glioma mouse model (91). Accordingly, it was demonstrated that in HMGB1-deficient tumors, different innate immune cells, including NK cells, have impaired ability to reach the tumor tissue in response to DNA alkylating agent treatments (92). In addition, HMGB1 can be released by NK cells and can stimulate NK cell chemotaxis through RAGE, thus further amplifying their response to tumors (93) and can also play an important role in the cross-talk between NK and DC, by promoting DC maturation (94, 95). Interestingly, HGMB1 can induce autophagy (96), which may control the regulation of the innate and adaptive immune responses contributing to enhance antigen processing and presentation (97).

Heat shock proteins are localized in most intracellular compartments where they act as molecular chaperone by supporting protein folding and transport across membranes. Several studies demonstrated an unusual HSP70 cell membrane localization on transformed tumor cells (98–100). As already mentioned, stressful conditions can cause HSPs mobilization to the plasma membrane, or their release from cells, thus acting as potent danger signals. In this respect, therapeutic treatments including radio and chemotherapy have been shown to produce an augmentation of HSP70 cell-surface expression on tumor cells (101, 102). Several studies have shown that membrane-bound HSP70 directly promotes NK cell mediated cytotoxicity *in vitro* (103, 104) and *in vivo* (105) thus, there is an increasing interest in the therapeutic potential of targeting HSP70. Interestingly, Elsner and colleagues have shown a synergistic potentiating effect of two stress-inducible immunological danger signals HSP70 and NKG2D ligands on cytotoxicity of human (106) and mouse NK cells (107), suggesting that the drug-mediated upregulation of activating ligands and HSP70 on the cancer cell surface might be an encouraging strategy aimed at promoting the antitumor NK cell responses. Moreover, several pieces of evidence demonstrate that extracellular-located HSPs can be associated to extracellular vesicles (108–112), and a number of chemotherapeutic agents, including etoposide (109), melphalan (110), cisplatin, and 5-fluorouracil (112), have been shown to stimulate an enhanced secretion of exosomes from different types of cancer cells. Notably, colon carcinoma-derived HSP70 associated to exosomes can stimulate NK cell migration and cytotoxic activity (108). In addition, we have recently demonstrated that HSP70 on the surface of MM-derived exosomes triggers NK cell-mediated IFN-γ production through a mechanism dependent on TLR2 (110).

## Direct Effects of Chemotherapy on NK Cell-Mediated Functions

Alterations of NK cell activities upon administration of chemotherapeutic drugs can be different in terms of cytotoxicity and immunoregulatory activity; indeed, standard chemotherapeutic protocols used in the treatment of cancer patients mainly suppress NK cell-mediated killing against cancer cells and their cytokine production. However, several studies aimed at analyzing the NK cell behavior in patients undergoing cytotoxic chemotherapy have demonstrated different and variable effects depending on both the type and the dose of the drug used.

In this regard, by producing IFN-γ, NK cells induce CD8^+^ T cells to become CTLs, and also help to differentiate CD4^+^ T cells toward a Th1 response. Moreover, NK cell-derived cytokines might also regulate antitumor antibody production by B cells. Thus, therapeutic strategies able to preserve NK functions in cancer patients are of pivotal importance, particularly those eligible for monoclonal antibody-based treatments. In this context, metronomic low cyclophosphamide (CTX) regimen was shown to potently stimulate NK functions in terms of cytokine production and antitumor immunity (18). A number of drugs, including bortezomib, genotoxic agents, and epigenetic drugs, exert immunosuppressive effects at high concentrations, whereas at sub-lethal doses, they can render tumor cells more immunogenic without affecting the immune cell activity (113). As an example, low doses of bortezomib capable of stimulating NK cell activating ligand expression on MM (22, 52), do not alter NK cell degranulation against sensitive targets (52). In another study, low concentrations of bortezomib reduced IFN-γ production without affecting NK cell cytotoxicity (114). Moreover, a combination of bortezomib with exogenous cytokine treatment enhanced the cytotoxic effects of NK cells against cancer cells in two different models (115, 116). The treatment of NK cells with sub-lethal doses of doxorubicin, able to upregulate NKG2D and DNAM-1 ligands on MM cells, does not change the capacity of NK cell to degranulate in response to target cells, as well as the ability to produce IFN-γ (34). Although the wide range of HDACi, structurally different from each other, can have both stimulatory and inhibitory effects on immune cell function, the most of them (i.e., romidepsin, vorinostat, TSA, and VPA) have been shown to suppress NK cell activity at therapeutically relevant concentrations (117–119). However, some reports describe a beneficial effect on NK cells as for the narrow-spectrum HDACi entinostat that can increase NKG2D expression on NK cells without affecting their cytotoxic activity (120). Furthermore, a recent study demonstrates that the HDACi panobinostat has the capability to potentiate the antitumor effects of trastuzumab by stimulating the antibody-dependent cell-mediated cytotoxicity (ADCC) mediated by NK cells (121). Regarding the DNTMi decitabine and 5-azacytidine, treatment of NK cells leads to increased reactivity toward different tumor cells (122, 123), while another study describes that 5-azacytidine exposure compromises their activity in AML and MDS patients (124).

Immunomodulatory drugs (lenalidomide, pomalidomide, and thalidomide) exert strong immunomodulatory effects involving both innate and adaptive immunity. In particular, these compounds activate both NK and T cells by inducing their proliferation, cytokine production, and cytotoxic activity (125) and promising clinical trials have been reported their use for the treatment of hematological malignancies, such as myeloma, lymphoma, and leukemia, as well as of solid tumors (126–128). Interestingly, Lagrue and colleagues demonstrated that lenalidomide enhances NK cell response (IFN-γ production and cytotoxicity) by augmenting actin remodeling, thus rendering them able to respond to lower densities of activating ligands on tumor cells (126). Furthermore, lenalidomide has synergistic effects on NK cell functions when used in combination with monoclonal antibodies able to promote ADCC that are already approved in therapeutic protocols, such as rituximab or elotuzumab (129, 130); indeed, novel strategies in the treatment of MM combines the use of lenalidomide and the anti-inhibitory KIR antibody (IPH2101) (131, 132).

## Conclusion

**Figure 1:**
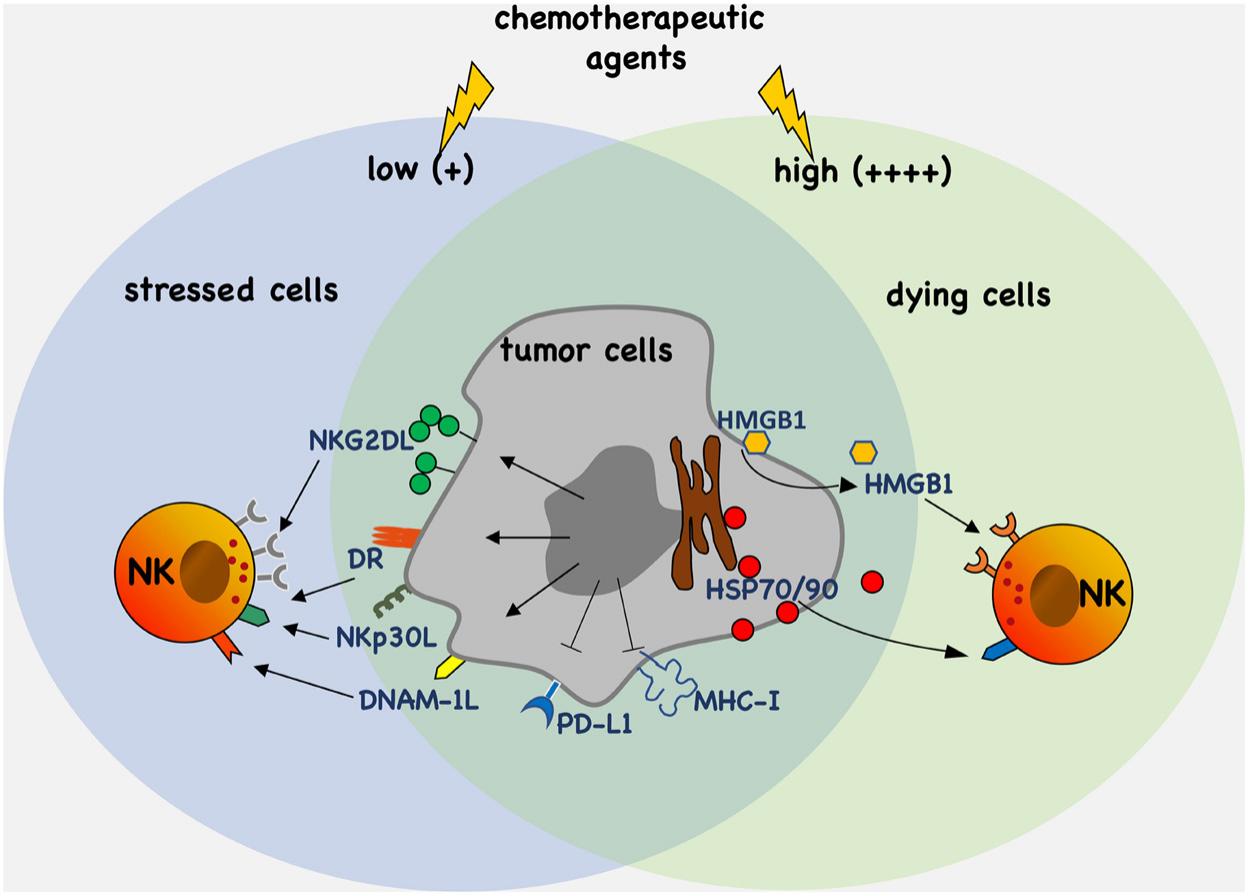
Antitumor efficacy of chemotherapy. Chemotherapeutic agents activate molecular pathways eliciting upregulation and/or the release of stress molecules that promote tumor cell recognition and elimination by natural killer (NK) cells. Moreover, chemotherapy can also downregulate the expression of ligands such as PD-L1 and major histocompatibility complex (MHC)-I of inhibitory receptors.

The modulation of the expression and/or the release of stress molecules has emerged as a new paradigm of the therapeutic possibilities associated with the use of chemotherapy (Figure 1). In this context, the characterization of novel drugs and regulatory pathways activated by cellular stress modifiers able to affect tumor growth and, at the same time, to improve the activities mediated by cytotoxic lymphocytes such as NK cells, will importantly contribute to the developing field of chemo-immunotherapy.

## Author Contributions

AZ, CF, CB, MC, ASantoni, and ASoriani contributed equally to writing and critically revised the paper.

## Conflict of Interest Statement

The authors declare that the research was conducted in the absence of any commercial or financial relationships that could be construed as a potential conflict of interest.

## References

[1] Caligiuri MA. Human natural killer cells. Blood (2008) 112(3):461–9. 10.1182/blood-2007-09-077438 18650461 PMC2481557

[2] Vivier E Raulet DH Moretta A Caligiuri MA Zitvogel L Lanier LL Innate or adaptive immunity? The example of natural killer cells. Science (2011) 331(6013):44–9. 10.1126/science.1198687 21212348 PMC3089969

[3] Lanier LL. NK cell recognition. Annu Rev Immunol (2005) 23:225–74. 10.1146/annurev.immunol.23.021704.115526 15771571

[4] Parham P. MHC class I molecules and KIRs in human history, health and survival. Nat Rev Immunol (2005) 5(3):201–14. 10.1038/nri1570 15719024

[5] Stanietsky N Simic H Arapovic J Toporik A Levy O Novik A The interaction of TIGIT with PVR and PVRL2 inhibits human NK cell cytotoxicity. Proc Natl Acad Sci U S A (2009) 106(42):17858–63. 10.1073/pnas.0903474106 19815499 PMC2764881

[6] Vivier E Tomasello E Baratin M Walzer T Ugolini S. Functions of natural killer cells. Nat Immunol (2008) 9(5):503–10. 10.1038/ni1582 18425107

[7] Krzewski K Coligan JE Human NK cell lytic granules and regulation of their exocytosis. Front Immunol (2012) 3:335 10.3389/fimmu.2012.00335 23162553 PMC3494098

[8] Voskoboinik I Smyth MJ Trapani JA. Perforin-mediated target-cell death and immune homeostasis. Nat Rev Immunol (2006) 6(12):940–52. 10.1038/nri1983 17124515

[9] Wallin RP Screpanti V Michaelsson J Grandien A Ljunggren HG. Regulation of perforin-independent NK cell-mediated cytotoxicity. Eur J Immunol (2003) 33(10):2727–35. 10.1002/eji.200324070 14515256

[10] Smyth MJ Cretney E Kelly JM Westwood JA Street SE Yagita H Activation of NK cell cytotoxicity. Mol Immunol (2005) 42(4):501–10. 10.1016/j.molimm.2004.07.034 15607806

[11] Alvarez IB Pasquinelli V Jurado JO Abbate E Musella RM de la Barrera SS Role played by the programmed death-1-programmed death ligand pathway during innate immunity against *Mycobacterium tuberculosis*. J Infect Dis (2010) 202(4):524–32. 10.1086/654932 20617899

[12] Norris S Coleman A Kuri-Cervantes L Bower M Nelson M Goodier MR. PD-1 expression on natural killer cells and CD8(+) T cells during chronic HIV-1 infection. Viral Immunol (2012) 25(4):329–32. 10.1089/vim.2011.0096 22742708

[13] Stojanovic A Fiegler N Brunner-Weinzierl M Cerwenka A CTLA-4 is expressed by activated mouse NK cells and inhibits NK Cell IFN-gamma production in response to mature dendritic cells. J Immunol (2014) 192(9):4184–91. 10.4049/jimmunol.1302091 24688023

[14] Zitvogel L Apetoh L Ghiringhelli F Andre F Tesniere A Kroemer G. The anticancer immune response: indispensable for therapeutic success? J Clin Invest (2008) 118(6):1991–2001. 10.1172/JCI35180 18523649 PMC2396905

[15] Zitvogel L Kepp O Kroemer G. Immune parameters affecting the efficacy of chemotherapeutic regimens. Nat Rev Clin Oncol (2011) 8(3):151–60. 10.1038/nrclinonc.2010.223 21364688

[16] Zitvogel L Apetoh L Ghiringhelli F Kroemer G. Immunological aspects of cancer chemotherapy. Nat Rev Immunol (2008) 8(1):59–73. 10.1038/nri2216 18097448

[17] Shurin MR Naiditch H Gutkin DW Umansky V Shurin GV. ChemoImmunoModulation: immune regulation by the antineoplastic chemotherapeutic agents. Curr Med Chem (2012) 19(12):1792–803. 10.2174/092986712800099785 22414087

[18] Bracci L Schiavoni G Sistigu A Belardelli F. Immune-based mechanisms of cytotoxic chemotherapy: implications for the design of novel and rationale-based combined treatments against cancer. Cell Death Differ (2014) 21(1):15–25. 10.1038/cdd.2013.67 23787994 PMC3857622

[19] Abruzzese MP Bilotta MT Fionda C Zingoni A Soriani A Vulpis E Inhibition of bromodomain and extra-terminal (BET) proteins increases NKG2D ligand MICA expression and sensitivity to NK cell-mediated cytotoxicity in multiple myeloma cells: role of cMYC-IRF4-miR-125b interplay. J Hematol Oncol (2016) 9(1):134. 10.1186/s13045-016-0362-2 27903272 PMC5131470

[20] Fionda C Malgarini G Soriani A Zingoni A Cecere F Iannitto ML Inhibition of glycogen synthase kinase-3 increases NKG2D ligand MICA expression and sensitivity to NK cell-mediated cytotoxicity in multiple myeloma cells: role of STAT3. J Immunol (2013) 190(12):6662–72. 10.4049/jimmunol.1201426 23686482

[21] Fionda C Soriani A Malgarini G Iannitto ML Santoni A Cippitelli M. Heat shock protein-90 inhibitors increase MHC class I-related chain A and B ligand expression on multiple myeloma cells and their ability to trigger NK cell degranulation. J Immunol (2009) 183(7):4385–94. 10.4049/jimmunol.0901797 19748980

[22] Soriani A Zingoni A Cerboni C Iannitto ML Ricciardi MR Di Gialleonardo V ATM-ATR-dependent up-regulation of DNAM-1 and NKG2D ligands on multiple myeloma cells by therapeutic agents results in enhanced NK-cell susceptibility and is associated with a senescent phenotype. Blood (2009) 113(15):3503–11. 10.1182/blood-2008-08-173914 19098271

[23] Cao G Wang J Zheng X Wei H Tian Z Sun R. Tumor therapeutics work as stress inducers to enhance tumor sensitivity to natural killer (NK) cell cytolysis by up-regulating NKp30 ligand B7-H6. J Biol Chem (2015) 290(50):29964–73. 10.1074/jbc.M115.674010 26472927 PMC4705966

[24] Jinushi M Vanneman M Munshi NC Tai YT Prabhala RH Ritz J MHC class I chain-related protein A antibodies and shedding are associated with the progression of multiple myeloma. Proc Natl Acad Sci U S A (2008) 105(4):1285–90. 10.1073/pnas.0711293105 18202175 PMC2234130

[25] Zhu Z Lu X Jiang L Sun X Zhou H Jia Z STAT3 signaling pathway is involved in decitabine induced biological phenotype regulation of acute myeloid leukemia cells. Am J Transl Res (2015) 7(10):1896–907. 26692933 PMC4656766

[26] Tang KF He CX Zeng GL Wu J Song GB Shi YS Induction of MHC class I-related chain B (MICB) by 5-aza-2’-deoxycytidine. Biochem Biophys Res Commun (2008) 370(4):578–83. 10.1016/j.bbrc.2008.03.131 18395517

[27] Rohner A Langenkamp U Siegler U Kalberer CP Wodnar-Filipowicz A. Differentiation-promoting drugs up-regulate NKG2D ligand expression and enhance the susceptibility of acute myeloid leukemia cells to natural killer cell-mediated lysis. Leuk Res (2007) 31(10):1393–402. 10.1016/j.leukres.2007.02.020 17391757

[28] Hogg SJ Vervoort SJ Deswal S Ott CJ Li J Cluse LA BET-bromodomain inhibitors engage the host immune system and regulate expression of the immune checkpoint ligand PD-L1. Cell Rep (2017) 18(9):2162–74. 10.1016/j.celrep.2017.02.011 28249162 PMC5340981

[29] Zhu H Bengsch F Svoronos N Rutkowski MR Bitler BG Allegrezza MJ BET bromodomain inhibition promotes anti-tumor immunity by suppressing PD-L1 expression. Cell Rep (2016) 16(11):2829–37. 10.1016/j.celrep.2016.08.032 27626654 PMC5177024

[30] Shi J Tricot GJ Garg TK Malaviarachchi PA Szmania SM Kellum RE Bortezomib down-regulates the cell-surface expression of HLA class I and enhances natural killer cell-mediated lysis of myeloma. Blood (2008) 111(3):1309–17. 10.1182/blood-2007-03-078535 17947507 PMC2214736

[31] Gorgun G Samur MK Cowens KB Paula S Bianchi G Anderson JE Lenalidomide enhances immune checkpoint blockade-induced immune response in multiple myeloma. Clin Cancer Res (2015) 21(20):4607–18. 10.1158/1078-0432.CCR-15-0200 25979485 PMC4609232

[32] Giuliani M Janji B Berchem G. Activation of NK cells and disruption of PD-L1/PD-1 axis: two different ways for lenalidomide to block myeloma progression. Oncotarget (2017) 8(14):24031–44. 10.18632/oncotarget.15234 28199990 PMC5410361

[33] Gasser S Orsulic S Brown EJ Raulet DH. The DNA damage pathway regulates innate immune system ligands of the NKG2D receptor. Nature (2005) 436(7054):1186–90. 10.1038/nature03884 15995699 PMC1352168

[34] Soriani A Iannitto ML Ricci B Fionda C Malgarini G Morrone S Reactive oxygen species- and DNA damage response-dependent NK cell activating ligand upregulation occurs at transcriptional levels and requires the transcriptional factor E2F1. J Immunol (2014) 193(2):950–60. 10.4049/jimmunol.1400271 24913980

[35] Lam AR Le Bert N Ho SS Shen YJ Tang ML Xiong GM RAE1 ligands for the NKG2D receptor are regulated by STING-dependent DNA sensor pathways in lymphoma. Cancer Res (2014) 74(8):2193–203. 10.1158/0008-5472.CAN-13-1703 24590060 PMC4229084

[36] Fine JH Chen P Mesci A Allan DS Gasser S Raulet DH Chemotherapy-induced genotoxic stress promotes sensitivity to natural killer cell cytotoxicity by enabling missing-self recognition. Cancer Res (2010) 70(18):7102–13. 10.1158/0008-5472.CAN-10-1316 20823164 PMC3108335

[37] Venkataraman GM Suciu D Groh V Boss JM Spies T. Promoter region architecture and transcriptional regulation of the genes for the MHC class I-related chain A and B ligands of NKG2D. J Immunol (2007) 178(2):961–9. 10.4049/jimmunol.178.2.961 17202358

[38] Nice TJ Coscoy L Raulet DH. Posttranslational regulation of the NKG2D ligand Mult1 in response to cell stress. J Exp Med (2009) 206(2):287–98. 10.1084/jem.20081335 19171762 PMC2646581

[39] Diermayr S Himmelreich H Durovic B Mathys-Schneeberger A Siegler U Langenkamp U NKG2D ligand expression in AML increases in response to HDAC inhibitor valproic acid and contributes to allorecognition by NK-cell lines with single KIR-HLA class I specificities. Blood (2008) 111(3):1428–36. 10.1182/blood-2007-07-101311 17993609

[40] Shi P Yin T Zhou F Cui P Gou S Wang C. Valproic acid sensitizes pancreatic cancer cells to natural killer cell-mediated lysis by upregulating MICA and MICB via the PI3K/Akt signaling pathway. BMC Cancer (2014) 14:370. 10.1186/1471-2407-14-370 24885711 PMC4076062

[41] Armeanu S Bitzer M Lauer UM Venturelli S Pathil A Krusch M Natural killer cell-mediated lysis of hepatoma cells via specific induction of NKG2D ligands by the histone deacetylase inhibitor sodium valproate. Cancer Res (2005) 65(14):6321–9. 10.1158/0008-5472.CAN-04-4252 16024634

[42] Kato N Tanaka J Sugita J Toubai T Miura Y Ibata M Regulation of the expression of MHC class I-related chain A, B (MICA, MICB) via chromatin remodeling and its impact on the susceptibility of leukemic cells to the cytotoxicity of NKG2D-expressing cells. Leukemia (2007) 21(10):2103–8. 10.1038/sj.leu.2404862 17625602

[43] Pfeiffer MM Burow H Schleicher S Handgretinger R Lang P. Influence of histone deacetylase inhibitors and DNA-methyltransferase inhibitors on the NK cell-mediated lysis of pediatric B-lineage leukemia. Front Oncol (2013) 3:99. 10.3389/fonc.2013.00099 23641363 PMC3638146

[44] Fiegler N Textor S Arnold A Rolle A Oehme I Breuhahn K Downregulation of the activating NKp30 ligand B7-H6 by HDAC inhibitors impairs tumor cell recognition by NK cells. Blood (2013) 122(5):684–93. 10.1182/blood-2013-02-482513 23801635

[45] Wu X Tao Y Hou J Meng X Shi J. Valproic acid upregulates NKG2D ligand expression through an ERK-dependent mechanism and potentially enhances NK cell-mediated lysis of myeloma. Neoplasia (2012) 14(12):1178–89. 10.1593/neo.121236 23308050 PMC3540943

[46] Yang H Lan P Hou Z Guan Y Zhang J Xu W Histone deacetylase inhibitor SAHA epigenetically regulates miR-17-92 cluster and MCM7 to upregulate MICA expression in hepatoma. Br J Cancer (2015) 112(1):112–21. 10.1038/bjc.2014.547 25393367 PMC4453603

[47] Fionda C Abruzzese MP Zingoni A Cecere F Vulpis E Peruzzi G The IMiDs targets IKZF-1/3 and IRF4 as novel negative regulators of NK cell-activating ligands expression in multiple myeloma. Oncotarget (2015) 6(27):23609–30. 10.18632/oncotarget.4603 26269456 PMC4695140

[48] Bellucci R Martin A Bommarito D Wang K Hansen SH Freeman GJ Interferon-gamma-induced activation of JAK1 and JAK2 suppresses tumor cell susceptibility to NK cells through upregulation of PD-L1 expression. Oncoimmunology (2015) 4(6):e1008824 10.1080/2162402X.2015.1008824 26155422 PMC4485824

[49] Ho V Lim TS Lee J Steinberg J Szmyd R Tham M TLR3 agonist and Sorafenib combinatorial therapy promotes immune activation and controls hepatocellular carcinoma progression. Oncotarget (2015) 6(29):27252–66. 10.18632/oncotarget.4583 26287667 PMC4694987

[50] Soriani A Borrelli C Ricci B Molfetta R Zingoni A Fionda C p38 MAPK differentially controls NK activating ligands at transcriptional and post-transcriptional level on multiple myeloma cells. Oncoimmunology (2017) 6(1):e1264564 10.1080/2162402X.2016.1264564 28197392 PMC5283620

[51] Acebes-Huerta A Lorenzo-Herrero S Folgueras AR Huergo-Zapico L Lopez-Larrea C Lopez-Soto A Drug-induced hyperploidy stimulates an antitumor NK cell response mediated by NKG2D and DNAM-1 receptors. Oncoimmunology (2016) 5(2):e1074378 10.1080/2162402X.2015.1074378 27057443 PMC4801427

[52] Niu C Jin H Li M Zhu S Zhou L Jin F Low-dose bortezomib increases the expression of NKG2D and DNAM-1 ligands and enhances induced NK and gammadelta T cell-mediated lysis in multiple myeloma. Oncotarget (2017) 8(4):5954–64. 10.18632/oncotarget.13979 27992381 PMC5351604

[53] Soriani A Fionda C Ricci B Iannitto ML Cippitelli M Santoni A. Chemotherapy-elicited upregulation of NKG2D and DNAM-1 ligands as a therapeutic target in multiple myeloma. Oncoimmunology (2013) 2(12):e26663. 10.4161/onci.26663 24498552 PMC3912005

[54] Zingoni A Vulpis E Nardone I Soriani A Fionda C Cippitelli M Targeting NKG2D and NKp30 ligands shedding to improve NK cell-based immunotherapy. Crit Rev Immunol (2016) 36(6):445–60. 10.1615/CritRevImmunol.2017020166 28845754

[55] Zingoni A Cecere F Vulpis E Fionda C Molfetta R Soriani A Genotoxic stress induces senescence-associated ADAM10-dependent release of NKG2D MIC ligands in multiple myeloma cells. J Immunol (2015) 195(2):736–48. 10.4049/jimmunol.1402643 26071561

[56] Lin X Huang M Xie F Zhou H Yang J Huang Q. Gemcitabine inhibits immune escape of pancreatic cancer by down regulating the soluble ULBP2 protein. Oncotarget (2016) 7(43):70092–9. 10.18632/oncotarget.11780 27602753 PMC5342537

[57] Raneros AB Puras AM Rodriguez RM Colado E Bernal T Anguita E Increasing TIMP3 expression by hypomethylating agents diminishes soluble MICA, MICB and ULBP2 shedding in acute myeloid leukemia, facilitating NK cell-mediated immune recognition. Oncotarget (2017) 8(19):31959–76. 10.18632/oncotarget.16657 28404876 PMC5458262

[58] Elrod HA Sun SY. Modulation of death receptors by cancer therapeutic agents. Cancer Biol Ther (2008) 7(2):163–73. 10.4161/cbt.7.2.5335 18059181

[59] Kim YH Park JW Lee JY Kwon TK. Sodium butyrate sensitizes TRAIL-mediated apoptosis by induction of transcription from the DR5 gene promoter through Sp1 sites in colon cancer cells. Carcinogenesis (2004) 25(10):1813–20. 10.1093/carcin/bgh188 15142888

[60] Nakata S Yoshida T Horinaka M Shiraishi T Wakada M Sakai T. Histone deacetylase inhibitors upregulate death receptor 5/TRAIL-R2 and sensitize apoptosis induced by TRAIL/APO2-L in human malignant tumor cells. Oncogene (2004) 23(37):6261–71. 10.1038/sj.onc.1207830 15208660

[61] Hirai S Endo S Saito R Hirose M Ueno T Suzuki H Antitumor effects of a sirtuin inhibitor, tenovin-6, against gastric cancer cells via death receptor 5 up-regulation. PLoS One (2014) 9(7):e102831. 10.1371/journal.pone.0102831 25033286 PMC4102575

[62] Guo F Sigua C Tao J Bali P George P Li Y Cotreatment with histone deacetylase inhibitor LAQ824 enhances Apo-2L/tumor necrosis factor-related apoptosis inducing ligand-induced death inducing signaling complex activity and apoptosis of human acute leukemia cells. Cancer Res (2004) 64(7):2580–9. 10.1158/0008-5472.CAN-03-2629 15059915

[63] Shankar S Singh TR Fandy TE Luetrakul T Ross DD Srivastava RK. Interactive effects of histone deacetylase inhibitors and TRAIL on apoptosis in human leukemia cells: involvement of both death receptor and mitochondrial pathways. Int J Mol Med (2005) 16(6):1125–38. 10.3892/ijmm.16.6.1125 16273296

[64] Fandy TE Shankar S Ross DD Sausville E Srivastava RK. Interactive effects of HDAC inhibitors and TRAIL on apoptosis are associated with changes in mitochondrial functions and expressions of cell cycle regulatory genes in multiple myeloma. Neoplasia (2005) 7(7):646–57. 10.1593/neo.04655 16026644 PMC1501425

[65] Insinga A Monestiroli S Ronzoni S Gelmetti V Marchesi F Viale A Inhibitors of histone deacetylases induce tumor-selective apoptosis through activation of the death receptor pathway. Nat Med (2005) 11(1):71–6. 10.1038/nm0205-233a 15619634

[66] Lundqvist A Abrams SI Schrump DS Alvarez G Suffredini D Berg M Bortezomib and depsipeptide sensitize tumors to tumor necrosis factor-related apoptosis-inducing ligand: a novel method to potentiate natural killer cell tumor cytotoxicity. Cancer Res (2006) 66(14):7317–25. 10.1158/0008-5472.CAN-06-0680 16849582

[67] Lundqvist A Yokoyama H Smith A Berg M Childs R. Bortezomib treatment and regulatory T-cell depletion enhance the antitumor effects of adoptively infused NK cells. Blood (2009) 113(24):6120–7. 10.1182/blood-2008-11-190421 19202127 PMC2699233

[68] Liu X Yue P Chen S Hu L Lonial S Khuri FR The proteasome inhibitor PS-341 (bortezomib) up-regulates DR5 expression leading to induction of apoptosis and enhancement of TRAIL-induced apoptosis despite up-regulation of c-FLIP and survivin expression in human NSCLC cells. Cancer Res (2007) 67(10):4981–8. 10.1158/0008-5472.CAN-06-4274 17510429

[69] Kabore AF Sun J Hu X McCrea K Johnston JB Gibson SB. The TRAIL apoptotic pathway mediates proteasome inhibitor induced apoptosis in primary chronic lymphocytic leukemia cells. Apoptosis (2006) 11(7):1175–93. 10.1007/s10495-006-8048-9 16699949

[70] He Q Huang Y Sheikh MS. Proteasome inhibitor MG132 upregulates death receptor 5 and cooperates with Apo2L/TRAIL to induce apoptosis in Bax-proficient and -deficient cells. Oncogene (2004) 23(14):2554–8. 10.1038/sj.onc.1207351 14691451

[71] Yoshida T Shiraishi T Nakata S Horinaka M Wakada M Mizutani Y Proteasome inhibitor MG132 induces death receptor 5 through CCAAT/enhancer-binding protein homologous protein. Cancer Res (2005) 65(13):5662–7. 10.1158/0008-5472.CAN-05-0693 15994939

[72] Wu GS Burns TF McDonald ER III Jiang W Meng R Krantz ID KILLER/DR5 is a DNA damage-inducible p53-regulated death receptor gene. Nat Genet (1997) 17(2):141–3. 10.1038/ng1097-141 9326928

[73] Takimoto R El-Deiry WS. Wild-type p53 transactivates the KILLER/DR5 gene through an intronic sequence-specific DNA-binding site. Oncogene (2000) 19(14):1735–43. 10.1038/sj.onc.1203489 10777207

[74] Guan B Yue P Clayman GL Sun SY. Evidence that the death receptor DR4 is a DNA damage-inducible, p53-regulated gene. J Cell Physiol (2001) 188(1):98–105. 10.1002/jcp.1101 11382926

[75] Liu X Yue P Khuri FR Sun SY. p53 upregulates death receptor 4 expression through an intronic p53 binding site. Cancer Res (2004) 64(15):5078–83. 10.1158/0008-5472.CAN-04-1195 15289308

[76] Shetty S Graham BA Brown JG Hu X Vegh-Yarema N Harding G Transcription factor NF-kappaB differentially regulates death receptor 5 expression involving histone deacetylase 1. Mol Cell Biol (2005) 25(13):5404–16. 10.1128/MCB.25.13.5404-5416.2005 15964798 PMC1156987

[77] Muller M Wilder S Bannasch D Israeli D Lehlbach K Li-Weber M p53 activates the CD95 (APO-1/Fas) gene in response to DNA damage by anticancer drugs. J Exp Med (1998) 188(11):2033–45. 10.1084/jem.188.11.2033 9841917 PMC2212386

[78] Sheikh MS Burns TF Huang Y Wu GS Amundson S Brooks KS el-Deiry WS: p53-dependent and -independent regulation of the death receptor KILLER/DR5 gene expression in response to genotoxic stress and tumor necrosis factor alpha. Cancer Res (1998) 58(8):1593–8. 9563466

[79] Gibson SB Oyer R Spalding AC Anderson SM Johnson GL. Increased expression of death receptors 4 and 5 synergizes the apoptosis response to combined treatment with etoposide and TRAIL. Mol Cell Biol (2000) 20(1):205–12. 10.1128/MCB.20.1.205-212.2000 10594023 PMC85076

[80] Nagane M Pan G Weddle JJ Dixit VM Cavenee WK Huang HJ. Increased death receptor 5 expression by chemotherapeutic agents in human gliomas causes synergistic cytotoxicity with tumor necrosis factor-related apoptosis-inducing ligand in vitro and in vivo. Cancer Res (2000) 60(4):847–53. 10706092

[81] Wen J Ramadevi N Nguyen D Perkins C Worthington E Bhalla K. Antileukemic drugs increase death receptor 5 levels and enhance Apo-2L-induced apoptosis of human acute leukemia cells. Blood (2000) 96(12):3900–6. 11090076

[82] Srivastava RK Mi QS Hardwick JM Longo DL. Deletion of the loop region of Bcl-2 completely blocks paclitaxel-induced apoptosis. Proc Natl Acad Sci U S A (1999) 96(7):3775–80. 10.1073/pnas.96.7.3775 10097113 PMC22370

[83] Eliopoulos AG Kerr DJ Herod J Hodgkins L Krajewski S Reed JC The control of apoptosis and drug resistance in ovarian cancer: influence of p53 and Bcl-2. Oncogene (1995) 11(7):1217–28. 7478541

[84] Siemer S Ornskov D Guerra B Boldyreff B Issinger OG. Determination of mRNA, and protein levels of p53, MDM2 and protein kinase CK2 subunits in F9 cells after treatment with the apoptosis-inducing drugs cisplatin and carboplatin. Int J Biochem Cell Biol (1999) 31(6):661–70. 10.1016/S1357-2725(99)00020-5 10404639

[85] Tolis C Peters GJ Ferreira CG Pinedo HM Giaccone G. Cell cycle disturbances and apoptosis induced by topotecan and gemcitabine on human lung cancer cell lines. Eur J Cancer (1999) 35(5):796–807. 10.1016/S0959-8049(98)00425-0 10505042

[86] Wang S Wang Z Boise L Dent P Grant S. Loss of the bcl-2 phosphorylation loop domain increases resistance of human leukemia cells (U937) to paclitaxel-mediated mitochondrial dysfunction and apoptosis. Biochem Biophys Res Commun (1999) 259(1):67–72. 10.1006/bbrc.1999.0669 10334917

[87] Garg AD Galluzzi L Apetoh L Baert T Birge RB Bravo-San Pedro JM Molecular and translational classifications of DAMPs in immunogenic cell death. Front Immunol (2015) 6:588. 10.3389/fimmu.2015.00588 26635802 PMC4653610

[88] Apetoh L Ghiringhelli F Tesniere A Obeid M Ortiz C Criollo A Toll-like receptor 4-dependent contribution of the immune system to anticancer chemotherapy and radiotherapy. Nat Med (2007) 13(9):1050–9. 10.1038/nm1622 17704786

[89] Chen T Guo J Han C Yang M Cao X. Heat shock protein 70, released from heat-stressed tumor cells, initiates antitumor immunity by inducing tumor cell chemokine production and activating dendritic cells via TLR4 pathway. J Immunol (2009) 182(3):1449–59. 10.4049/jimmunol.182.3.1449 19155492

[90] Lotze MT Tracey KJ. High-mobility group box 1 protein (HMGB1): nuclear weapon in the immune arsenal. Nat Rev Immunol (2005) 5(4):331–42. 10.1038/nri1594 15803152

[91] Wu J Waxman DJ. Metronomic cyclophosphamide eradicates large implanted GL261 gliomas by activating antitumor Cd8+ T-cell responses and immune memory. Oncoimmunology (2015) 4(4):e1005521. 10.1080/2162402X.2015.1005521 26137402 PMC4485826

[92] Guerriero JL Ditsworth D Catanzaro JM Sabino G Furie MB Kew RR DNA alkylating therapy induces tumor regression through an HMGB1-mediated activation of innate immunity. J Immunol (2011) 186(6):3517–26. 10.4049/jimmunol.1003267 21300822 PMC3066027

[93] Parodi M Pedrazzi M Cantoni C Averna M Patrone M Cavaletto M Natural Killer (NK)/melanoma cell interaction induces NK-mediated release of chemotactic High Mobility Group Box-1 (HMGB1) capable of amplifying NK cell recruitment. Oncoimmunology (2015) 4(12):e1052353. 10.1080/2162402X.2015.1052353 26587323 PMC4635845

[94] Semino C Angelini G Poggi A Rubartelli A. NK/iDC interaction results in IL-18 secretion by DCs at the synaptic cleft followed by NK cell activation and release of the DC maturation factor HMGB1. Blood (2005) 106(2):609–16. 10.1182/blood-2004-10-3906 15802534

[95] Semino C Ceccarelli J Lotti LV Torrisi MR Angelini G Rubartelli A. The maturation potential of NK cell clones toward autologous dendritic cells correlates with HMGB1 secretion. J Leukoc Biol (2007) 81(1):92–9. 10.1189/jlb.0306172 16997859

[96] Tang D Kang R Livesey KM Cheh CW Farkas A Loughran P Endogenous HMGB1 regulates autophagy. J Cell Biol (2010) 190(5):881–92. 10.1083/jcb.200911078 20819940 PMC2935581

[97] Valdor R Macian F. Autophagy and the regulation of the immune response. Pharmacol Res (2012) 66(6):475–83. 10.1016/j.phrs.2012.10.003 23063674 PMC3508673

[98] Multhoff G Botzler C Wiesnet M Muller E Meier T Wilmanns W A stress-inducible 72-kDa heat-shock protein (HSP72) is expressed on the surface of human tumor cells, but not on normal cells. Int J Cancer (1995) 61(2):272–9. 10.1002/ijc.2910610222 7705958

[99] Gehrmann M Schmetzer H Eissner G Haferlach T Hiddemann W Multhoff G Membrane-bound heat shock protein 70 (Hsp70) in acute myeloid leukemia: a tumor specific recognition structure for the cytolytic activity of autologous NK cells. Haematologica (2003) 88(4):474–6. 12681978

[100] Pfister K Radons J Busch R Tidball JG Pfeifer M Freitag L Patient survival by Hsp70 membrane phenotype: association with different routes of metastasis. Cancer (2007) 110(4):926–35. 10.1002/cncr.22864 17580361

[101] Kleinjung T Arndt O Feldmann HJ Bockmuhl U Gehrmann M Zilch T Heat shock protein 70 (Hsp70) membrane expression on head-and-neck cancer biopsy-a target for natural killer (NK) cells. Int J Radiat Oncol Biol Phys (2003) 57(3):820–6. 10.1016/S0360-3016(03)00629-1 14529789

[102] Gehrmann M Pfister K Hutzler P Gastpar R Margulis B Multhoff G. Effects of antineoplastic agents on cytoplasmic and membrane-bound heat shock protein 70 (Hsp70) levels. Biol Chem (2002) 383(11):1715–25. 10.1515/BC.2002.192 12530536

[103] Gehrmann M Schonberger J Zilch T Rossbacher L Thonigs G Eilles C Retinoid- and sodium-butyrate-induced decrease in heat shock protein 70 membrane-positive tumor cells is associated with reduced sensitivity to natural killer cell lysis, growth delay, and altered growth morphology. Cell Stress Chaperones (2005) 10(2):136–46. 10.1379/CSC-88R1.1 16038410 PMC1176472

[104] Gross C Holler E Stangl S Dickinson A Pockley AG Asea AA An Hsp70 peptide initiates NK cell killing of leukemic blasts after stem cell transplantation. Leuk Res (2008) 32(4):527–34. 10.1016/j.leukres.2007.03.027 17543383

[105] Multhoff G Pfister K Botzler C Jordan A Scholz R Schmetzer H Adoptive transfer of human natural killer cells in mice with severe combined immunodeficiency inhibits growth of Hsp70-expressing tumors. Int J Cancer (2000) 88(5):791–7. 10.1002/1097-0215(20001201)88:5<791::AID-IJC17>3.0.CO;2-I 11072250

[106] Elsner L Flugge PF Lozano J Muppala V Eiz-Vesper B Demiroglu SY The endogenous danger signals HSP70 and MICA cooperate in the activation of cytotoxic effector functions of NK cells. J Cell Mol Med (2010) 14(4):992–1002. 10.1111/j.1582-4934.2009.00677.x 20569278 PMC3823130

[107] Elsner L Muppala V Gehrmann M Lozano J Malzahn D Bickeboller H The heat shock protein HSP70 promotes mouse NK cell activity against tumors that express inducible NKG2D ligands. J Immunol (2007) 179(8):5523–33. 10.4049/jimmunol.179.8.5523 17911639

[108] Gastpar R Gehrmann M Bausero MA Asea A Gross C Schroeder JA Heat shock protein 70 surface-positive tumor exosomes stimulate migratory and cytolytic activity of natural killer cells. Cancer Res (2005) 65(12):5238–47. 10.1158/0008-5472.CAN-04-3804 15958569 PMC1785299

[109] Lv LH Wan YL Lin Y Zhang W Yang M Li GL Anticancer drugs cause release of exosomes with heat shock proteins from human hepatocellular carcinoma cells that elicit effective natural killer cell antitumor responses in vitro. J Biol Chem (2012) 287(19):15874–85. 10.1074/jbc.M112.340588 22396543 PMC3346092

[110] Vulpis E Cecere F Molfetta R Soriani A Fionda C Peruzzi G Genotoxic stress modulates the release of exosomes from multiple myeloma cells capable of activating NK cell cytokine production: role of HSP70/TLR2/NF-kB axis. Oncoimmunology (2017) 6(3):e1279372. 10.1080/2162402X.2017.1279372 28405503 PMC5384384

[111] Lancaster GI Febbraio MA. Exosome-dependent trafficking of HSP70: a novel secretory pathway for cellular stress proteins. J Biol Chem (2005) 280(24):23349–55. 10.1074/jbc.M502017200 15826944

[112] Gobbo J Marcion G Cordonnier M Dias AMM Pernet N Hammann A Restoring anticancer immune response by targeting tumor-derived exosomes with a HSP70 peptide aptamer. J Natl Cancer Inst (2016) 108(3):djv330. 10.1093/jnci/djv330 26598503

[113] Pellom ST Jr Dudimah DF Thounaojam MC Sayers TJ Shanker A. Modulatory effects of bortezomib on host immune cell functions. Immunotherapy (2015) 7(9):1011–22. 10.2217/imt.15.66 26325610 PMC4648628

[114] Armeanu S Krusch M Baltz KM Weiss TS Smirnow I Steinle A Direct and natural killer cell-mediated antitumor effects of low-dose bortezomib in hepatocellular carcinoma. Clin Cancer Res (2008) 14(11):3520–8. 10.1158/1078-0432.CCR-07-4744 18519785

[115] Wang X Feng X Wang J Shao N Ji C Ma D Bortezomib and IL-12 produce synergetic anti-multiple myeloma effects with reduced toxicity to natural killer cells. Anticancer Drugs (2014) 25(3):282–8. 10.1097/CAD.0000000000000058 24300915

[116] Khan T Stauffer JK Williams R Hixon JA Salcedo R Lincoln E Proteasome inhibition to maximize the apoptotic potential of cytokine therapy for murine neuroblastoma tumors. J Immunol (2006) 176(10):6302–12. 10.4049/jimmunol.176.10.6302 16670342

[117] Ni L Wang L Yao C Ni Z Liu F Gong C The histone deacetylase inhibitor valproic acid inhibits NKG2D expression in natural killer cells through suppression of STAT3 and HDAC3. Sci Rep (2017) 7:45266. 10.1038/srep45266 28338101 PMC5364405

[118] Ogbomo H Michaelis M Kreuter J Doerr HW Cinatl J Jr. Histone deacetylase inhibitors suppress natural killer cell cytolytic activity. FEBS Lett (2007) 581(7):1317–22. 10.1016/j.febslet.2007.02.045 17349632

[119] Kelly-Sell MJ Kim YH Straus S Benoit B Harrison C Sutherland K The histone deacetylase inhibitor, romidepsin, suppresses cellular immune functions of cutaneous T-cell lymphoma patients. Am J Hematol (2012) 87(4):354–60. 10.1002/ajh.23112 22367792 PMC3638752

[120] Zhu S Denman CJ Cobanoglu ZS Kiany S Lau CC Gottschalk SM The narrow-spectrum HDAC inhibitor entinostat enhances NKG2D expression without NK cell toxicity, leading to enhanced recognition of cancer cells. Pharm Res (2015) 32(3):779–92. 10.1007/s11095-013-1231-0 24203492 PMC4014531

[121] Medon M Vidacs E Vervoort SJ Li J Jenkins MR Ramsbottom KM HDAC inhibitor panobinostat engages host innate immune defenses to promote the tumoricidal effects of trastuzumab in HER2+ tumors. Cancer Res (2017) 77(10):2594–606. 10.1158/0008-5472.CAN-16-2247 28249907

[122] Schmiedel BJ Arelin V Gruenebach F Krusch M Schmidt SM Salih HR. Azacytidine impairs NK cell reactivity while decitabine augments NK cell responsiveness toward stimulation. Int J Cancer (2011) 128(12):2911–22. 10.1002/ijc.25635 20960460

[123] Sohlberg E Pfefferle A Andersson S Baumann BC Hellstrom-Lindberg E Malmberg KJ. Imprint of 5-azacytidine on the natural killer cell repertoire during systemic treatment for high-risk myelodysplastic syndrome. Oncotarget (2015) 6(33):34178–90. 10.18632/oncotarget.6213 26497557 PMC4741444

[124] Schonefeldt C Sockel K Wehner R Sopper S Wolf D Wermke M Azacytidine impairs NK cell activity in AML and MDS patients undergoing MRD-based pre-emptive treatment after allogeneic stem cell transplantation. Blood Cancer J (2013) 3:e136 10.1038/bcj.2013.35 23995045 PMC3763388

[125] Sehgal K Das R Zhang L Verma R Deng Y Kocoglu M Clinical and pharmacodynamic analysis of pomalidomide dosing strategies in myeloma: impact of immune activation and cereblon targets. Blood (2015) 125(26):4042–51. 10.1182/blood-2014-11-611426 25869284 PMC4481593

[126] Lagrue K Carisey A Morgan DJ Chopra R Davis DM. Lenalidomide augments actin remodeling and lowers NK-cell activation thresholds. Blood (2015) 126(1):50–60. 10.1182/blood-2015-01-625004 26002964 PMC4551357

[127] Acebes-Huerta A Huergo-Zapico L Gonzalez-Rodriguez AP Fernandez-Guizan A Payer AR Lopez-Soto A Lenalidomide induces immunomodulation in chronic lymphocytic leukemia and enhances antitumor immune responses mediated by NK and CD4 T cells. Biomed Res Int (2014) 2014:265840. 10.1155/2014/265840 25313353 PMC4182694

[128] Shortt J Hsu AK Johnstone RW. Thalidomide-analogue biology: immunological, molecular and epigenetic targets in cancer therapy. Oncogene (2013) 32(36):4191–202. 10.1038/onc.2012.599 23318436

[129] Sidaway P Haematological cancer: rituximab enhances responses to lenalidomide. Nat Rev Clin Oncol (2017) 14(2):70 10.1038/nrclinonc.2016.209 27958295

[130] Gormley NJ Ko CW Deisseroth A Nie L Kaminskas E Kormanik N FDA drug approval: elotuzumab in combination with lenalidomide and dexamethasone for the treatment of relapsed or refractory multiple myeloma. Clin Cancer Res (2017). 10.1158/1078-0432.CCR-16-2870 28249893

[131] Benson DM Jr Cohen AD Jagannath S Munshi NC Spitzer G Hofmeister CC A phase I trial of the anti-KIR antibody IPH2101 and lenalidomide in patients with relapsed/refractory multiple myeloma. Clin Cancer Res (2015) 21(18):4055–61. 10.1158/1078-0432.CCR-15-0304 25999435 PMC4573800

[132] Nijhof IS Lammerts van Bueren JJ van Kessel B Andre P Morel Y Lokhorst HM Daratumumab-mediated lysis of primary multiple myeloma cells is enhanced in combination with the human anti-KIR antibody IPH2102 and lenalidomide. Haematologica (2015) 100(2):263–8. 10.3324/haematol.2014.117531 25510242 PMC4803142

